# Evaluation of Laccase Activities by Three Newly Isolated Fungal Species in Submerged Fermentation With Single or Mixed Lignocellulosic Wastes

**DOI:** 10.3389/fmicb.2021.682679

**Published:** 2021-06-07

**Authors:** Mei-Ling Han, Jing Yang, Ze-Yang Liu, Chun-Rui Wang, Si-Yu Chen, Ning Han, Wen-Yao Hao, Qi An, Yu-Cheng Dai

**Affiliations:** ^1^College of Life Sciences, Langfang Normal University, Langfang, China; ^2^Technical Innovation Center for Utilization of Edible and Medicinal Fungi in Hebei Province, Langfang, China; ^3^Edible and Medicinal Fungi Research and Development Center of Universities, Colleges in Hebei Province, Langfang, China; ^4^Beijing Advanced Innovation Center for Tree Breeding By Molecular Design, Beijing Forestry University, Beijing, China

**Keywords:** laccase activity, co-culture, single lignocellulosic waste, mixed lignocellulosic wastes, submerged fermentation

## Abstract

Three newly isolated fungal species, namely, *Cerrena unicolor* Han 849, *Lenzites betulina* Han 851, and *Schizophyllum commune* Han 881, isolated from their native habitats in Wulingshan National Nature Reserve of Hebei Province of northern China, were screened for laccase production with single or mixed lignocellulosic wastes. *C. unicolor* Han 849 was found to express the highest levels of laccase with single or mixed lignocellulosic wastes compared with *L. betulina* Han 851 and *S. commune* Han 881. The highest laccase activity from the mixed fungal culture of *C. unicolor* Han 849 and *S. commune* Han 881 or *L. betulina* Han 851 on *Firmiana platanifolia* was 1,373.12 ± 55.93 and 1,144.85 ± 34.97 U/L, respectively, higher than that from other tested conditions. *L. betulina* Han 851 or *S. commune* Han 881 mixed with other species was also helpful for accelerating laccase secretion due to reach maximum enzyme activity quickly. The treatment of mixing different species, including the mixture of two or three species, was obviously conducive to the improvement of laccase activity on *Firmiana platanifolia*. These results revealed that the fungal co-culture and the mixed lignocellulosic wastes contribute to the improvement of laccase activities and enhance laccase activities within a short period. These findings would be helpful for providing a new method for rapid production of low-cost laccase and for optimization of integrated industrial laccase production.

## Introduction

The rapid development of agriculture and forestry is conducive to economic development and environmental protection. Meanwhile, it also brings some environmental problems, and the most important is agricultural and forestry residues. Also, if agricultural and forestry waste is burned, it will cause even more serious air pollution. Agricultural and forestry waste is an important kind of lignocellulosic biomass. Lignocellulosic biomass, versatile and most abundant natural renewable resources, have attracted more attention and considerable interest due to their abilities to convert into green second-generation energy fuels and valued industrial chemicals *via* the various bio-treatment process of lignocellulosic biomass (Haldar et al., 2016; Pinar et al., 2017; An et al., 2020b; Gaikwad and Meshram, 2020). Using various lignocellulosic wastes, such as corncob, cottonseed hull, bamboos, coffee shell, and tree leaves, to produce enzymes (especially laccase) had been widely investigated in recent years due to the low-cost and vast amounts of lignocellulosic wastes (Lizardi-Jimenez et al., 2019; Thamvithayakorn et al., 2019; Wang et al., 2019; Agrawal and Verma, 2020; Atilano-Camino et al., 2020; Pinheiro et al., 2020; Xu et al., 2020). Laccase (EC 1.10.3.2, *p*-benzenediol: oxygen oxidoreductase) belongs to a family of copper oxidases and widely distributes in various higher plants, fungi, bacteria, and some insects (Zhang et al., 2020). Due to the ability of laccase to oxidize a wide range of phenolic and non-phenolic compounds, laccase can be used in the various biotechnological process, including materials science, bioremediation, biofuels, fiber modified, nanobiotechnology, biosensor, food chemistry, paper and pulp industry, and biodegradation (Yang et al., 2017; Becker and Wittmann, 2019; Bilal et al., 2019; Singh and Arya, 2019; Unuofin et al., 2019a; Wang et al., 2019; Zerva et al., 2019; An et al., 2020a). White-rot fungi, belonging to basidiomycetes, are well known for their essential role in degrading lignocellulose in their native habitats due to their ability to secrete various extracellular ligninolytic enzymes (Thamvithayakorn et al., 2019). Among ligninolytic enzymes, laccase is the oldest and important enzyme that could degrade lignin. Meanwhile, white-rot fungi are considered the famous laccase producer, and almost all species among them have the capacity for secreting laccase to some extent (Agrawal et al., 2018; An et al., 2019; Huang et al., 2019; Lira-Perez et al., 2020).

Due to its wide application in numerous fields of biotechnology, more and more researchers have shown great interest in all aspects of laccase (Ma and Ruan, 2015; Wang et al., 2015; An et al., 2016a, 2018; Rodriguez et al., 2019; Zhang et al., 2020). Meanwhile, large amounts of laccase with low cost were required for the widespread use of laccase in the various biotechnological process (Osma et al., 2011; An et al., 2018; Zerva et al., 2019; Zhang et al., 2020). To enhance the laccase production and reduce the cost of producing laccase, optimization of fermentation condition, fermentation method, and developing new productivity strains are very effective methods. Optimization of fermentation condition was mainly included the following categories: (1) category, concentration, and proportion of carbon and nitrogen sources (Kannaiyan et al., 2012; Birhanli and Yeşilada, 2013; Zhou et al., 2014; Han et al., 2017, 2020b; Palazzolo et al., 2019; Thamvithayakorn et al., 2019; Unuofin et al., 2019b); (2) metal ions, such as copper ion, ferrous ion, manganese ion, silver ion, zinc ion, and magnesium ion (Hu et al., 2014; Yang et al., 2016; Zhuo et al., 2017; Xu et al., 2018; An et al., 2020a); (3) temperature and pH (Hu et al., 2014; Kaira et al., 2015; Mazlan and Abu Hanifah, 2017; Saoudi et al., 2017; Bettin et al., 2019); and (4) secondary metabolites, e.g., ferulic acid and veratrol (Janusz et al., 2015; Yang et al., 2016). The fermentation method was divided into solid-state fermentation, submerged fermentation, and unconventional solid-state fermentation combined with agitated submerged fermentation (An et al., 2016b). The advantage of solid-state fermentation is that it is closer to the natural environment of fungi and more energy-efficient, such as using less water. The advantage of submerged fermentation is that it is more manageable and durable, and industrial applications were more preferred to submerged fermentation. Different species or different strains belonging to the same species affected the laccase activity significantly (Janusz et al., 2015; An et al., 2020a). Thus, developing new strains with the capacity of producing laccase is very meaningful work.

Previous studies had indicated the capacity of secreting laccase by *Cerrena unicolor* (Mazur et al., 2015; Songulashvili et al., 2015; Wang et al., 2017; Zhang et al., 2018). However, the ability to secrete laccase from *Lenzites betulinus* and *Schizophyllum* sp. had not been reported. Co-culture of fungi has been studied in recent years and has a good effect on secreting laccase, whereas almost all studies investigated the white-rot fungi combined with mycete or yeasts to produce laccase (Rodriguez et al., 2019; Lira-Perez et al., 2020; Zhang et al., 2020). The effect of using two or three white-rot fungi combined with each other to produce laccase was rarely investigated (Kuhar et al., 2015; Vibha and Negi, 2018). Most studies had used only one lignocellulosic material to explore the effect on laccase secreted by fungi, and very few had considered using a mixture of two lignocellulosic materials to explore the effect on laccase secretion by fungi (Lallawmsanga, Leo et al., 2019; Thamvithayakorn et al., 2019; Unuofin et al., 2019b; Gaikwad and Meshram, 2020; Xu et al., 2020). However, evaluation of laccase activities from *C. unicolor*, *Lenzites betulinus*, and *Schizophyllum* sp. had not been investigated till now, not only the effects of co-culture of these species but also the effects of mixed lignocellulosic wastes on their enzyme production. Under the circumstances, the laccase production capacity of three newly isolated fungal species with single or mixed lignocellulosic wastes was analyzed in the present work. Also, the effect of co-culture of these species on laccase activity was investigated at the same time. The results were contributed to provide new methods to improve laccase production and obtain low-cost laccase.

## Materials and Methods

### Culture of Microorganisms

Three fungal species, *C. unicolor* Han 849, *Lenzites betulina* Han 851, and *Schizophyllum* sp. Han 881, were newly isolated from their native habitats in Wulingshan National Nature Reserve of Hebei Province of northern China. These species were isolated and purified on complete yeast medium (CYM), and the pure cultures of these species were preserved on malt extract agar medium at 4°C in the College of Life Science, Langfang Normal University.

### Collection of Lignocellulosic Wastes

Lignocellulosic wastes, *Pinus tabuliformis* and *Firmiana platanifolia*, were obtained from Chengde city (Hebei province, China). All these lignocellulosic wastes were air-dried and milled to a particle size of between 20 and 60 mesh.

### Microbial Culture and Inoculum Preparation

To activate the used three strains, all microorganisms were incubated on CYM for 7 days at 26°C. Then, five inoculants holed by a perforator with a diameter of 5 mm from corresponding Petri plates were placed in 250-ml flasks containing 100 ml of CYM without agar. All flasks were cultured in an oscillating culture shaker with a speed of 150 rpm at 26°C. After 7 days, the mycelium pellets in the Erlenmeyer flask were homogenized by modular homogenizer HFJ-10 (Tianjin HengAo Technology Co., Ltd.) at 5,000 rpm for 2 min. Also, the homogenized liquid was used as an inoculum.

### Time Course of Laccase Activity

Erlenmeyer flasks (250 ml) containing 2-g single lignocellulosic waste or mixed with two kinds of lignocellulosic wastes were soaked with 100 ml of solution (1.5-g monopotassium phosphate dissolved into 1 L of deionized water) and autoclaved at 121°C for 30 min. All flasks were sterilized at 121°C for 30 min. After autoclaving, each Erlenmeyer flasks was added to the 3 ml of homogenized inoculum according to the list in Table 1. Then, all flasks were transferred to a rotary shaker (26°C, 150 rpm) for various fermentation times.

**TABLE 1:** Description of each component of the experimental group.

Species	Lignocellulosic wastes	Solution (ml)	Homogenized inoculum
*Cerrena unicolor* Han 849	*Pinus tabuliformis* 2 g	100	3 ml
	*Firmiana platanifolia* 2 g	100	3 ml
	*Pinus tabuliformis* 1 g and *Firmiana platanifolia* 1 g	100	3 ml
*Lenzites betulina* Han 851	*Pinus tabuliformis* 2 g	100	3 ml
	*Firmiana platanifolia* 2 g	100	3 ml
	*Pinus tabuliformis* 1 g and *Firmiana platanifolia* 1 g	100	3 ml
*Schizophyllum* sp. Han 881	*Pinus tabuliformis* 2 g	100	3 ml
	*Firmiana platanifolia* 2 g	100	3 ml
	*Pinus tabuliformis* 1 g and *Firmiana platanifolia* 1 g	100	3 ml
*C. unicolor* Han 849 and *L. betulina* Han 851	*Pinus tabuliformis* 2 g	100	1.5 ml of Han 849 and 1.5 ml of Han 851
	*Firmiana platanifolia* 2 g	100	1.5 ml of Han 849 and 1.5 ml of Han 851
	*Pinus tabuliformis* 1 g and *Firmiana platanifolia* 1 g	100	1.5 ml of Han 849 and 1.5 ml of Han 851
*C. unicolor* Han 849 and *Schizophyllum* sp. Han 881	*Pinus tabuliformis* 2 g	100	1.5 ml of Han 849 and 1.5 ml of Han 881
	*Firmiana platanifolia* 2 g	100	1.5 ml of Han 849 and 1.5 ml of Han 881
	*Pinus tabuliformis* 1 g and *Firmiana platanifolia* 1 g	100	1.5 ml of Han 849 and 1.5 ml of Han 881
*L. betulina* Han 851 and *Schizophyllum* sp. Han 881	*Pinus tabuliformis* 2 g	100	1.5 ml of Han 851 and 1.5 ml of Han 881
	*Firmiana platanifolia* 2 g	100	1.5 ml of Han 851 and 1.5 ml of Han 881
	*Pinus tabuliformis* 1 g and *Firmiana platanifolia* 1 g	100	1.5 ml of Han 851 and 1.5 ml of Han 881
*C. unicolor* Han 849, *L. betulina* Han 851 and *Schizophyllum* sp. Han 881	*Pinus tabuliformis* 2 g	100	1.0 ml of Han 849, 1.0 ml of Han 851 and 1.0 ml of Han 881
	*Firmiana platanifolia* 2 g	100	1.0 ml of Han 849, 1.0 ml of Han 851 and 1.0 ml of Han 881
	*Pinus tabuliformis* 1 g and *Firmiana platanifolia* 1 g	100	1.0 m of Han 849, 1.0 ml of Han 851 and 1.0 ml of Han 881

### Preparation of Crude Enzyme

To obtain the crude enzyme solution, the liquid in the Erlenmeyer flask at different fermentation times was filtered through a filter paper. The obtained filtrate was centrifuged at 4°C with a speed of 12,000 rpm for 20 min, and the supernatant was used for the determination of laccase activity.

### Assay of Laccase Activity

Laccase activity was assayed using 2,2′-azinobis-[3-ethyltiazoline-6-sulfonate] as substrate and monitored by an iMark^TM^ microplate absorbance reader (Bio-Rad, Hercules, CA, United States). The details of the reaction mixture involving the amount of each component and the process of determining laccase at 415 nm were referred to in the method of Han et al. (2020b). One unit of laccase activity was defined as the amount of enzyme required to oxidize 1 μmol of 2,2′-azinobis-[3-ethyltiazoline-6-sulfonate] per minute (ϵ_415_ = 3.16 × 10^4^ M^–1^ cm^–1^).

### Data Analysis

Results in this study were the mean values of triplicate experiments. Two-way analysis of variance followed by the Tukey *post-hoc* test was applied to examine the effects of lignocellulosic wastes and species on laccase activities according to An et al. (2020a, b), and the analysis of statistical tests was performed by SPSS software version 22.0 (PROC GLM, IBM SPSS software version 22.0, Armonk, NY, United States). All colorful figures were generated by Origin 2016 software (OriginLab Corporation, Northampton, MA, United States).

### Identification of the Fungus *Schizophyllum* sp. Han 881

Mycelia of *Schizophyllum* sp. Han 881 used for DNA extraction was grown on CYM medium for 7 days. An appropriate amount of mycelium was scraped by a sterile surgical blade, transferred into the EP tube, and ground by a TGrinder OSE-Y30 Tissue Homogenizer (Tiangen Biotech Co., Ltd., Beijing, China). The genomic DNA of *Schizophyllum* sp. Han 881 was extracted by cetyltrimethylammonium bromide rapid plant genome extraction kit-DN14 (Aidlab Biotechnologies Co., Ltd., Beijing, China) according to the instructions with some modifications (Han et al., 2016, 2020a). The primer pairs and PCR reaction schedule for amplifying the internally transcribed spacer regions of ribosomal DNA of Han 881 were referred to the method of Han et al. (2021b). The PCR products were sequenced with the same primer pairs and measured at Beijing Genomics Institute (Beijing, China). The sequence was analyzed and submitted to GenBank. Phylogenetic analysis followed Han et al. (2016). Maximum parsimony analysis was performed in PAUP^∗^ version 4.0b10 (Swofford, 2002). All characters were equally weighted, and gaps were treated as missing data. Trees were inferred using the heuristic search option with TBR branch swapping and 1,000 random sequence additions. Max trees were set to 5,000, branches of zero length were collapsed, and all parsimonious trees were saved. Clade robustness was assessed using a bootstrap analysis with 1,000 replicates (Felsenstein, 1985). Branches that received bootstrap values for MP greater than or equal to 75% were considered as significantly supported. Phylogenetic trees were visualized using Treeview (Page, 1996).

## Results

### Molecular Biological Results of Fungus *Schizophyllum* sp. Han 881

**FIGURE 1:**
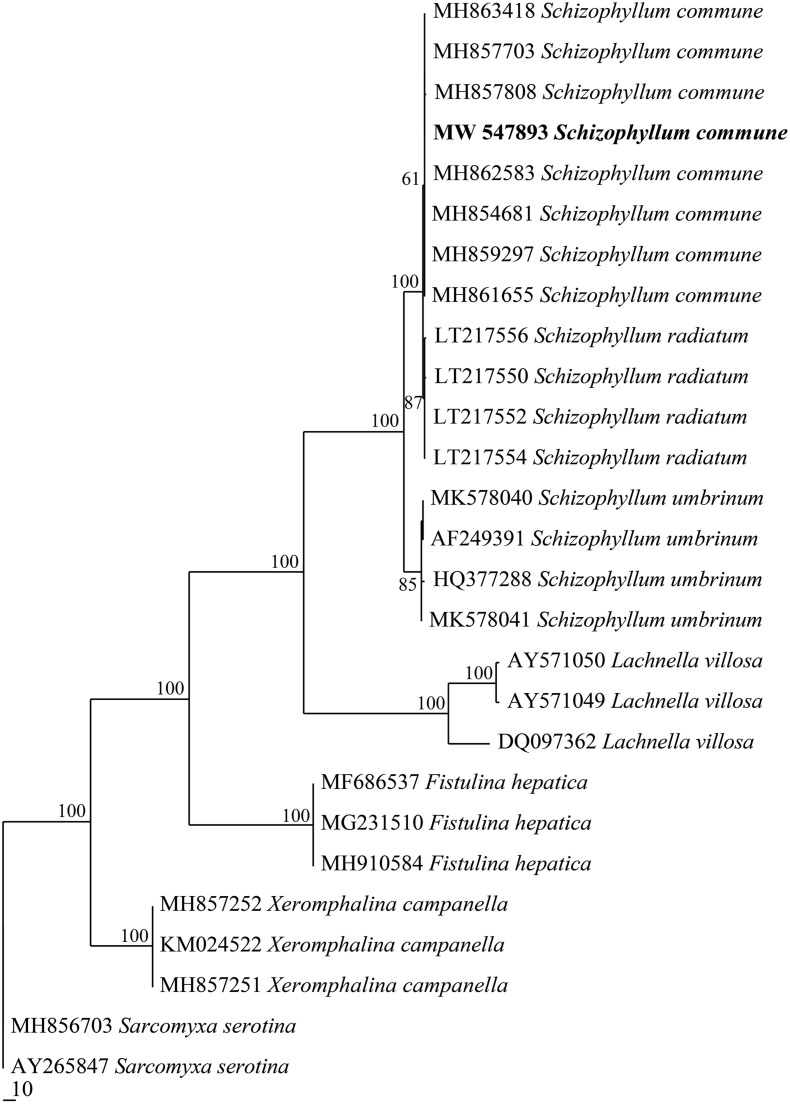
Maximum parsimony strict consensus tree illustrating the phylogeny of *Schizophyllum commune* and related species based on internally transcribed spacer sequence data. Branches are labeled with parsimony bootstrap proportions high than 50%.

The GenBank number of its sequence for Han 881 was MW 767989. The fungus *Schizophyllum* sp. Han 881 was grouped with samples of *Schizophyllum commune* downloaded from GenBank in the internally transcribed spacer phylogenetic tree (Figure 1) and then was identified as *S. commune*.

### Statistical Analysis Results

As shown in Table 2, the effect of species on laccase activity was significant (*P* < 0.001) during the whole process of submerged fermentation. Lignocellulosic wastes affected the laccase activity throughout the submerged fermentation phase (*P* < 0.001). Furthermore, the interaction of species and lignocellulosic wastes on laccase activity was significant in the whole fermentation stage (*P* < 0.001).

**TABLE 2:** Two-way ANOVA of variance to examine the effects of species, lignocellulosic wastes, and the interactions of species and lignocellulosic wastes on laccase activity.

Incubation Period (d)	Species	Lignocellulosic wastes	Species × lignocellulosic wastes
1	680.059***	606.774***	577.374***
2	1463.494***	2439.607***	733.093***
3	1677.145***	2447.436***	364.129***
4	2883.911***	4248.612***	734.370***
5	1339.770***	3504.977***	352.862***
6	1737.832***	3543.392***	387.125***
7	668.056***	2097.517***	181.140***
8	866.986***	3181.248***	299.093***
9	1010.451***	3565.245***	486.254***
10	1152.112***	4664.800***	740.748***

df = 6, 2, 12; ***P < 0.001.

### Evaluation of Laccase Activity From Single or Mixed Fungal Species on *Pinus tabuliformis*

**FIGURE 2:**
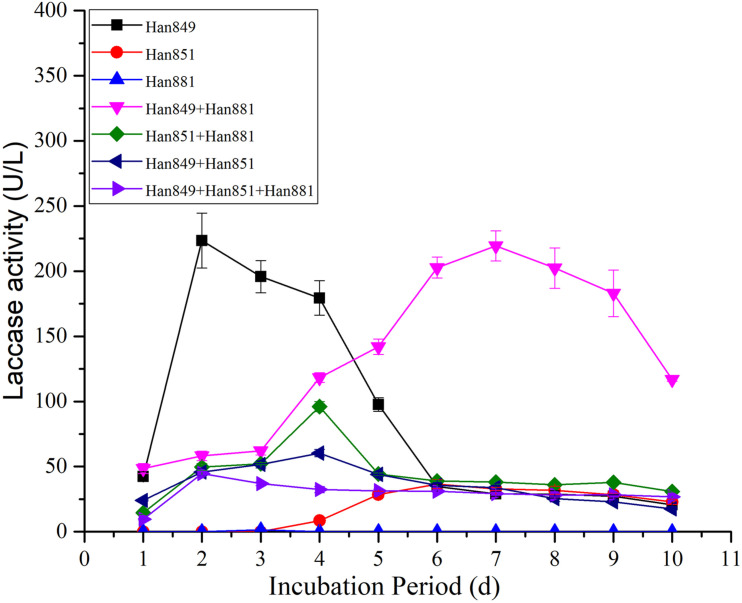
Laccase activity from single or mixed fungal species on *Pinus tabuliformis* in submerged fermentation.

In terms of the value of laccase activity on the first day, laccase activity values from *C. unicolor* Han 849, *L. betulina* Han 851, *S. commune* Han 881, a mixture of Han 849 and Han 881, a mixture of Han 851 and Han 881, a mixture of Han 849 and Han 851, and a mixture of Han 849, Han 851, and Han 881 were 42.29 ± 3.83, 0, 0, 48.52 ± 3.78, 14.47 ± 1.31, 24.01 ± 0.46, and 9.54 ± 0.46 U/L, respectively (Supplementary Tables 1–7). Based on this, a mixture of Han 849 and Han 881 was helpful to enhance the laccase activity. Laccase activity from *C. unicolor* Han 849 was 223.53 ± 21.06 U/L on the 2nd day, higher than others (Table 3). The first appearance time of laccase activity from *L. betulina* Han 851 was on the fourth day, and a corresponding value of laccase activity was only 8.54 ± 0.70 U/L (Supplementary Table 2). The laccase activity from *S. commune* Han 881 was only detected on the third day, which was only 1.51 ± 0.00 U/L (Supplementary Table 3). Maximum laccase activity from *C. unicolor* Han 849 was 223.53 ± 21.06 U/L on day 2, which was higher than that from *L. betulina* Han 851 (36.57 ± 3.39 U/L, day 6), *S. commune* Han 881 (1.51 ± 0.00 U/L, day 3), a mixture of Han 849 and Han 881 (219.41 ± 11.63 U/L, day 7), a mixture of Han 851 and Han 881 (96.04 ± 3.93 U/L, day 4), a mixture of Han 849 and Han 851 (60.38 ± 2.93 U/L, day 4), and a mixture of Han 849, Han 851, and Han 881 (44.71 ± 1.36 U/L, day 2), nearly 6.11, 148. 03−, 1. 02−, 2. 33−, 3. 70−, and 5.00-fold, respectively (Table 3). The enzyme production trend of *C. unicolor* Han 849, *S. commune* Han 881, a mixture of Han 851 and Han 881, a mixture of Han 849 and Han 851 (60.38 ± 2.93 U/L, day 4), and a mixture of Han 849, Han 851, and Han 881 (44.71 ± 1.36 U/L, day 2) was similar, and the maximum laccase activity appeared in the early fermentation stage (day ≤ 4). However, the trend of producing laccase from *C. unicolor* Han 849 and a mixture of Han 849, Han 851, and Han 881 was similar, and corresponding maximum laccase activity appeared in the intermediate stage of fermentation (day ≥ 6) (Figure 2). Compared with the single *L. betulina* Han 851 or *S. commune* Han 881, *L. betulina* Han 851 or *S. commune* Han 881 mixed with other species, e.g., a mixture of Han 849 and Han 881, a mixture of Han 851 and Han 881, a mixture of Han 849 and Han 851, and a mixture of Han 849, Han 851, and Han 881, were helpful for improving laccase activity based on the value of maximum laccase activity (Figure 2). Also, the time of maximum laccase activity from *L. betulina* Han 851 or *S. commune* Han 881 mixed with other species was earlier than that from single *L. betulina* Han 851 or *S. commune* Han 881 (Figure 2). Meanwhile, *L. betulina* Han 851 or *S. commune* Han 881 mixed with other species was also helpful for accelerating laccase secretion due to the first time laccase was detected (Figure 2).

**TABLE 3:** Maximum laccase production, Lignocellulosic wastes, and time of *Cerrena unicolor* Han 849, *Lenzites betulina* Han 851, and *Schizophyllum commune* Han 881.

Maximumlaccaseproduction(U/L)	Lignocellulosic wastes	Fungi species	Time (day)
223.53 ± 21.06	*Pinus tabuliformis*	Han 849	2
552.34 ± 49.14	*Firmiana platanifolia*	Han 849	3
876.23 ± 20.82	*Pinus tabuliformis* and *Firmiana platanifolia*	Han 849	4
36.57 ± 3.39	*Pinus tabuliformis*	Han 851	6
309.72 ± 12.53	*Firmiana platanifolia*	Han 851	7
136.23 ± 3.67	*Pinus tabuliformis* and *Firmiana platanifolia*	Han 851	4
1.51 ± 0.00	*Pinus tabuliformis*	Han 881	3
5.22 ± 0.35	*Firmiana platanifolia*	Han 881	7
3.32 ± 0.30	*Pinus tabuliformis* and *Firmiana platanifolia*	Han 881	8
219.41 ± 11.63	*Pinus tabuliformis*	Han 849 and Han 881	7
1,373.12 ± 55.93	*Firmiana platanifolia*	Han 849 and Han 881	6
785.61 ± 37.51	*Pinus tabuliformis* and *Firmiana platanifolia*	Han 849 and Han 881	6
96.04 ± 3.93	*Pinus tabuliformis*	Han 851 and Han 881	4
549.83 ± 12.42	*Firmiana platanifolia*	Han 851 and Han 881	5
183.34 ± 13.13	*Pinus tabuliformis* and *Firmiana platanifolia*	Han 851 and Han 881	7
60.38 ± 2.93	*Pinus tabuliformis*	Han 849 and Han 851	4
1,144.85 ± 34.97	*Firmiana platanifolia*	Han 849 and Han 851	10
390.30 ± 12.89	*Pinus tabuliformis* and *Firmiana platanifolia*	Han 849 and Han 851	8
44.71 ± 1.36	*Pinus tabuliformis*	Han 849, Han 851 and Han 881	2
774.96 ± 13.79	*Firmiana platanifolia*	Han 849, Han 851 and Han 881	10
274.46 ± 16.10	*Pinus tabuliformis* and *Firmiana platanifolia*	Han 849, Han 851 and Han 881	6

*Data are presented as mean ± standard deviation for triplicates and are expressed as U/L.*

### Evaluation of Laccase Activity From Single or Mixed Fungal Species on *Firmiana platanifolia*

**FIGURE 3:**
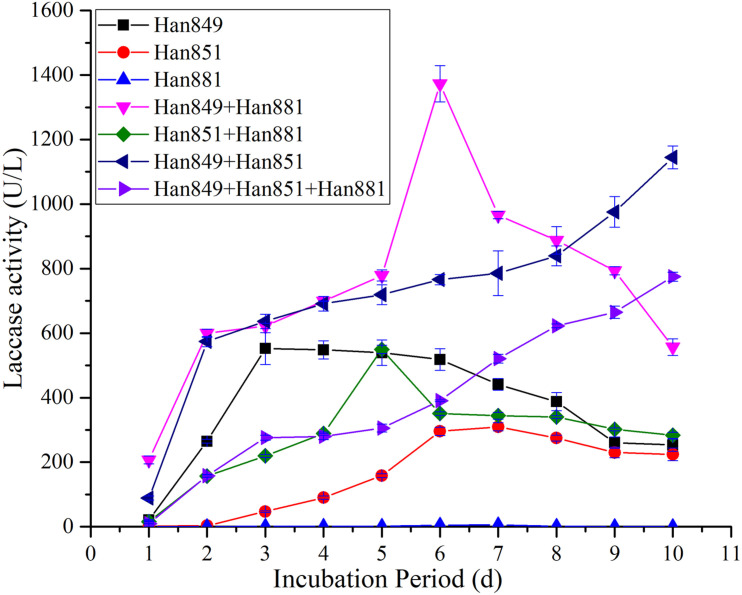
Laccase activity from single or mixed fungal species on *Firmiana platanifolia* in submerged fermentation.

Laccase activity values from *C. unicolor* Han 849, *L. betulina* Han 851, *S. commune* Han 881, a mixture of Han 849 and Han 881, a mixture of Han 851 and Han 881, a mixture of Han 849 and Han 851, and a mixture of Han 849, Han 851, and Han 881 were 20.90 ± 1.94, 2.01 ± 0.17, 0, 206.95 ± 12.14, 15.47 ± 0.97, 88.71 ± 6.19, and 9.34 ± 0.52 U/L, respectively, on the first day (Supplementary Tables 1–7). Obviously, laccase activity values from a mixture of Han 849 and Han 881 were nearly 9. 90−, 102. 96−, 13. 38−, 2. 33−, and 22.16-fold higher than that from *C. unicolor* Han 849, *L. betulina* Han 851, a mixture of Han 851 and Han 881, a mixture of Han 849 and Han 851, and a mixture of Han 849, Han 851, and Han 881, respectively. Maximum laccase activity values from a mixture of Han 849 and Han 881 (1,373.12 ± 55.93 U/L, day 6), which was higher than that from *C. unicolor* Han 849 (552.34 ± 49.14 U/L, day 3), *L. betulina* 851 (309.72 ± 12.53 U/L, day 7), *S. commune* Han 881 (5.22 ± 0.35 U/L, day 7), a mixture of Han 851 and Han 881 (549.83 ± 12.42 U/L, day 5), a mixture of Han 849 and Han 851 (1144.85 ± 34.97 U/L, day 10), and a mixture of Han 849, Han 851, and Han 881 (774.96 ± 13.79 U/L, day 10), nearly 2. 49−, 4. 43−, 263. 05−, 2. 50−, 1. 20−, and 1.77−fold, respectively (Table 3). Based on this, it was obvious that the treatment of mixing different species, whether the mixture of two or three species, was conducive to the improvement of laccase activity. Meanwhile, the treatment of mixing different species was helpful for occurring the continuous, higher, and stable laccase activity throughout the fermentation stage (Figure 3). Another, the capacity of secreting laccase by *C. unicolor* Han 849 was superior to *L. betulina* Han 851 and *S. commune* Han 881.

### Evaluation of Laccase Activity From Single or Mixed Fungal Species on a Mixture by *Pinus tabuliformis* and *Firmiana platanifolia*

**FIGURE 4:**
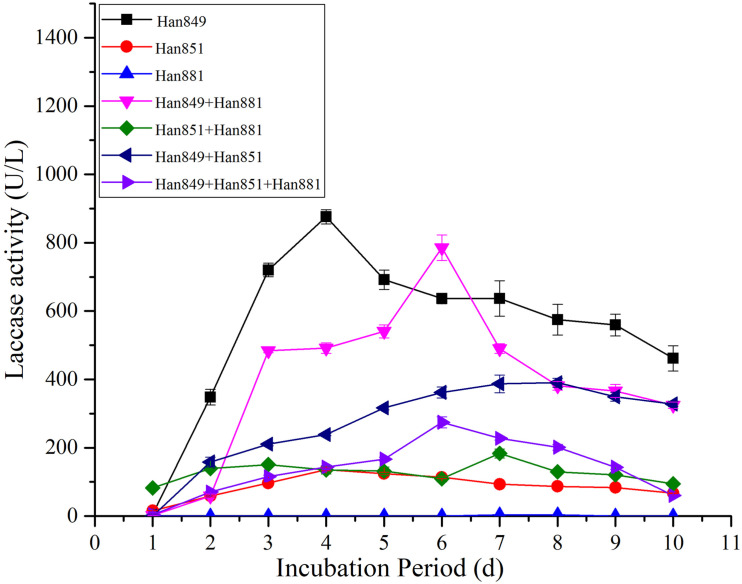
Laccase activity from single or mixed fungal species on *Pinus tabuliformis* and *Firmiana platanifolia* in submerged fermentation.

Laccase activity values from *C. unicolor* Han 849, *L. betulina* Han 851, *S. commune* Han 881, a mixture of Han 849 and Han 881, a mixture of Han 851 and Han 881, a mixture of Han 849 and Han 851, and a mixture of Han 849, Han 851, and Han 881 were 5.32 ± 0.17, 15.67 ± 0.52, 0.00, 1.51 ± 0.00, 82.38 ± 4.44, 0.00, and 3.72 ± 0.35 U/L on the first day (Supplementary Tables 1–7). Maximum laccase activity from *C. unicolor* Han 849 (876.23 ± 20.82 U/L, day 4), which was higher than that from *L. betulina* Han 851 (136.23 ± 3.67 U/L, day 4), *S. commune* Han 881 (3.32 ± 0.30 U/L, day 8), a mixture of Han 849 and Han 881 (785.61 ± 37.51 U/L, day 6), a mixture of Han 851 and Han 881 (183.34 ± 13.13 U/L, day 7), a mixture of Han 849 and Han 851 (390.30 ± 12.89 U/L, day 8), and a mixture of Han 849, Han 851, and Han 881 (274.46 ± 16.10 U/L, day 6), nearly 6. 43−, 263. 92−, 1. 12−, 4. 78−, 2. 25−, and 3.19-fold, respectively (Table 3). The enzyme production trend of *C. unicolor* Han 849 and *L. betulina* Han 851 was similar, and the maximum laccase activity appeared in the early fermentation stage (day ≤ 4). In contrast, the trend of producing laccase from *S. commune* Han 881, a mixture of Han 849 and Han 881, a mixture of Han 851 and Han 881, a mixture of Han 849 and Han 851, and a mixture of Han 849, Han 851, and Han 881 was similar, and corresponding maximum laccase activity appeared in the intermediate stage of fermentation (day ≥ 6) (Figure 4). Compared with the single *L. betulina* Han 851, *L. betulina* Han 851 mixed with other species, e.g., a mixture of Han 851 and Han 881, a mixture of Han 849 and Han 851, and a mixture of Han 849, Han 851, and Han 881, were helpful for improving laccase activity based on the value of maximum laccase activity (Figure 4). However, the time of maximum laccase activity from *L. betulina* Han 851 mixed with other species was later than that from the condition of single *L. betulina* Han 851 (Figure 4).

### Comparative of Laccase Activity From Single or Mixed Fungal Species on Different Lignocellulosic Wastes

Maximum laccase activity values from *C. unicolor* Han 849 on *P. tabuliformis*, *F. platanifolia*, and a mixture of *P. tabuliformis* and *F. platanifolia* were 223.53 ± 21.06, 552.34 ± 49.14, and 876.23 ± 20.82 U/L, respectively (Table 3). Obviously, the presence of mixed lignocellulosic wastes was a benefit for promoting the secretion of laccase by *C. unicolor* Han 849. Furthermore, a continuous and stable laccase activity from *C. unicolor* Han 849 could be achieved on *F. platanifolia*, and a mixture of *P. tabuliformis* and *F. platanifolia* (Figures 3, 4). Apart from *C. unicolor* Han 849, maximum laccase activity from other conditions on *F. platanifolia* was higher than that from *P. tabuliformis* or a mixture of *P. tabuliformis* and *F. platanifolia*. Maximum laccase activity from *L. betulina* Han 851 on *F. platanifolia* was 309.72 ± 12.53 U/L, nearly 8.47− and 2.27-fold higher than that on *P. tabuliformis* and a mixture of *P. tabuliformis* and *F. platanifolia*, respectively (Figures 2–4). Maximum laccase activity from the mixed fungal culture of Han 849 and Han 881 on *F. platanifolia* was 1,373.12 ± 55.93 U/L, nearly 6.26− and 1.75-fold higher than that on *P. tabuliformis* and a mixture of *P. tabuliformis* and *F. platanifolia*, respectively. Maximum laccase activity from the mixed fungal culture of Han 851 and Han 881 on *F. platanifolia* was 549.83 ± 12.42 U/L, nearly 5.73− and 3.00−fold higher than that on *P. tabuliformis* and a mixture of *P. tabuliformis* and *F. platanifolia*, respectively (Figures 2–4). Maximum laccase activity from the mixed fungal culture of Han 849 and Han 851 on *F. platanifolia* was 1,144.85 ± 34.97 U/L, nearly 18.96− and 2.93-fold higher than that on *P. tabuliformis* and a mixture of *P. tabuliformis* and *F. platanifolia*, respectively. Maximum laccase activity from the mixed fungal culture of Han 849, Han 851, and Han 881 on *F. platanifolia* was 774.96 ± 13.79 U/L, nearly 17.33− and 2.82-fold higher than that on *P. tabuliformis* and a mixture of *P. tabuliformis* and *F. platanifolia*, respectively (Figures 2–4). So, the mixed fungal culture of Han 849 with Han 851 or Han 881 on *F. platanifolia* was conducted to improving laccase activity compared with other conditions. Meanwhile, the laccase activity of either single species or mixed species on *P. tabuliformis* was lower than that on *F. platanifolia* or a mixture by *P. tabuliformis* and *F. platanifolia*.

## Discussion

Recent works have shown the ability of lignocellulosic biomass stimulating laccase production by basidiomycetes (Birhanli and Yeşilada, 2013; Zhou et al., 2014; Han et al., 2017; Palazzolo et al., 2019; Thamvithayakorn et al., 2019; Unuofin et al., 2019b; Han et al., 2020b, 2021a). Also, the selection of appropriate residues for fungus growth and target enzyme synthesis plays an important role in the development of efficient biotechnology (Elisashvili et al., 2008). Elisashvili et al. (2008) reported that laccase activity values from *Pleurotus ostreatus* IBB 8, *P. ostreatus* 2175, *Pleurotus tuberregium* IBB 624, *Lentinus edodes* IBB 123, *L. edodes* IBB 363, and *L. edodes* IBB 369 on tree leaves or wheat straw *via* solid-state fermentation were 7 ± 0.7 U/flask or 7 ± 0.8 U/flask, 15 ± 1.4 or 12 ± 1.2 U/flask, 20 ± 1.8 or 10 ± 1.0 U/flask, 57 ± 4.7 or 20 ± 1.5 U/flask, 52 ± 4.9 or 55 ± 5.1 U/flask, and 7 ± 0.7 or 38 ± 4.0 U/flask, respectively. The highest laccase activity values were 386 U/L for *Trametes trogii* incubated in a medium containing pulverized apricot seed shell and 1,216 U/L for *Trametes versicolor* grown in a medium containing pulverized bulrush in submerged fermentation (Birhanli and Yeşilada, 2013). The highest laccase from *Pseudolagarobasidium* sp. PP17-33 was 5.841 U/g using the oil palm decanter cake as materials for optimization of the production of enzymes through Plackett–Burman design (Thamvithayakorn et al., 2019). The optimal conditions for laccase production from *T. versicolor* were found at 35°C and 5 g/L of wheat bran as substrate, reaching approximately 200 U/ml on 11 days in submerged fermentation (Atilano-Camino et al., 2020). An et al. (2020b) reported that laccase production from *P. ostreatus* and *Flammulina velutipes* strains grown on cottonseed hull was better than that on corncob or poplar wood, and laccase production from *P. ostreatus* CCEF 89 grown on cottonseed hull, corncob, and poplar sawdust ranged from 61.38 ± 4.09 to 748.24 ± 9.53 U/L, 26.12 ± 2.28 to 699.12 ± 44.91 U/L, and 3.32 ± 0.30 to 509.75 ± 15.43 U/L, respectively. So, previous studies were mainly focused on the effect of single lignocellulosic biomass on laccase activity. Of course, some studies focused on the effect of different kinds of lignocellulosic biomass on laccase production secreted by fungi. However, no studies have been conducted on fermentation of laccase by using single coniferous trees, or broadleaf trees, or by mixing coniferous and broadleaf trees. Also, the effects of *P. tabuliformis* belonging to coniferous trees and *F. platanifolia* belonging to broadleaf trees on laccase activity secreted by three newly isolated species were investigated, and the effect of the mixture of *P. tabuliformis* and *F. platanifolia* on laccase activity was also studied. The results showed that the presence of a mixture by *P. tabuliformis* and *F. platanifolia* was a benefit for promoting the laccase activity by *C. unicolor* Han 849. Apart from *C. unicolor* Han 849, the effect of *F. platanifolia* was more contributed to tested fungi secreting laccase than the effect of *P. tabuliformis*.

Previous studies had indicated that different species or different strains belonging to the same species are an important factor affecting laccase activity (Lamia et al., 2017; Huang et al., 2019; An et al., 2020a,b; Han et al., 2020b). In other words, the biosynthetic potential of Basidiomycetes was highly dependent on the species of fungi (Han et al., 2021a). An et al. (2020b) reported that the capacity of secreting laccase of *P. ostreatus* strains was superior to *F. velutipes* strains due to the maximum laccase production on cottonseed hull, corncob, and poplar wood. A previous study indicated that maximum laccase activity of *P. ostreatus* CY 568 and CCEF 99 on poplar sawdust appeared on the fifth day and the nineth day (Han et al., 2020b). In this study, the capacity of secreting laccase from *C. unicolor* Han 849 was stronger than *L. betulinus* Han 851 and *S. commune* Han 881.

For the higher yield of ligninolytic enzymes, the cocultivation of *Phanerochaete chrysosporium* and *P. ostreatus* was investigated (Verma and Madamwar, 2002). Maximum laccase activity from the mixed fungal culture of *Trametes hirsuta* and *Phanerochaete* sp. was found to be 78.25 U/g with wheat bran:pulse husk:mustard peel (WB:PH:MP) in 2:2:1 ratio as substrate at pH 5.0 temperature 30°C and incubation time of 7 days (Vibha and Negi, 2018). Kuhar et al. (2015) reported that cocultivation of *Ganoderma lucidum* and *T. versicolor* was performed and showed remarkable enhancement of laccase activity. Similarly, *L. betulina* Han 851 or *S. commune* Han 881 mixed with other species was also helpful for accelerating laccase secretion in this study. Furthermore, it was obvious that the treatment of mixing different species, whether the mixture of two or three species, was conducive to the improvement of laccase activity on *F. platanifolia*.

## Conclusion

The effects of single or mixed lignocellulosic wastes and single or mixed fungal cultures were investigated in the present study. The presence of a mixture of *P. tabuliformis* and *F. platanifolia* was a benefit for promoting the laccase activity by *C. unicolor* Han 849. Apart from *C. unicolor* Han 849, the effect of *F. platanifolia* was more contributed to tested fungi secreting laccase than the effect of *P. tabuliformis*. The capacity of secreting laccase by *C. unicolor* Han 849 that was superior to *L. betulina* Han 851 and *S. commune* Han 881. *L. betulina* Han 851 or *S. commune* Han 881 mixed with other species was also helpful for accelerating laccase secretion. The treatment of mixing different species, including the mixture of two or three species, was obviously conducive to the improvement of laccase activity on *F. platanifolia*.

## Data Availability Statement

The datasets presented in this study can be found in online repositories. The names of the repository/repositories and accession number(s) can be found in the article/Supplementary Material.

## Author Contributions

M-LH, QA, and Y-CD: conceptualization. QA and M-LH: funding acquisition. M-LH, Z-YL, JY, C-RW, S-YC, NH, and W-YH: methodology. Z-YL, JY, C-RW, S-YC, NH, W-YH, and QA: data analysis. M-LH, W-YH, and QA: collect the materials. M-LH, JY, and C-RW: writing—original draft. QA and Y-CD: writing—review and editing. All authors contributed to the article and approved the submitted version.

## Conflict of Interest

The authors declare that the research was conducted in the absence of any commercial or financial relationships that could be construed as a potential conflict of interest.

## Supplementary Material

Supplementary Table 1 Laccase activity from *Cerrena unicolor* Han 849 grown on *Pinus tabuliformis*, *Firmiana platanifolia*, and a mixture by *Pinus tabuliformis* and *Firmiana platanifolia*.

Supplementary Table 2 Laccase activity from *Lenzites betulina* Han 851 grown on *Pinus tabuliformis*, *Firmiana platanifolia*, and a mixture by *Pinus tabuliformis* and *Firmiana platanifolia*.

Supplementary Table 3 Laccase activity from *Schizophyllum commune* Han 881 grown on *Pinus tabuliformis*, *Firmiana platanifolia*, and a mixture by *Pinus tabuliformis* and *Firmiana platanifolia*.

Supplementary Table 4 Laccase activity from *Cerrena unicolor* Han 849 and *Schizophyllum commune* Han 881 grown on *Pinus tabuliformis*, *Firmiana platanifolia*, and a mixture by *Pinus tabuliformis* and *Firmiana platanifolia*.

Supplementary Table 5 Laccase activity from *Lenzites betulina* Han 851 and *Schizophyllum commune* Han 881 grown on *Pinus tabuliformis*, *Firmiana platanifolia*, and a mixture by *Pinus tabuliformis* and *Firmiana platanifolia*.

Supplementary Table 6 Laccase activity from *Cerrena unicolor* Han 849 and *Lenzites betulina* Han 851 grown on *Pinus tabuliformis*, *Firmiana platanifolia*, and a mixture by *Pinus tabuliformis* and *Firmiana platanifolia*.

Supplementary Table 7 Laccase activity from *Cerrena unicolor* Han 849, *Lenzites betulina* Han 851 and *Schizophyllum commune* Han 881 grown on *Pinus tabuliformis*, *Firmiana platanifolia*, and a mixture by *Pinus tabuliformis* and *Firmiana platanifolia*.
