# Supplementary material for: Evaluation of Laccase Activities by Three Newly Isolated Fungal Species in Submerged Fermentation With Single or Mixed Lignocellulosic Wastes
Source: Front Microbiol. 2021 Jun 7;12:682679. doi: 10.3389/fmicb.2021.682679 (PMC8216501; doi:10.3389/fmicb.2021.682679)
Supplement: Supplementary Table 1 — Laccase activity from Cerrena unicolor Han 849 grown on Pinus tabuliformis, Firmiana platanifolia, and a mixture by Pinus tabuliformis and Firmiana platanifolia. [file Table_1.DOCX]

**Supplementary Table 1** Laccase activity from *Cerrena unicolor* Han 849 grown on *Pinus tabuliformis*, *Firmiana platanifolia*, and a mixture by *Pinus tabuliformis* and *Firmiana platanifolia*.

| Day | Laccase of *Cerrena unicolor* Han 849 (U/L) | | |
| --- | --- | --- | --- |
|  | *Pinus tabuliformis* | *Firmiana platanifolia* | A Mixture by *Pinus tabuliformis* and *Firmiana platanifolia* |
| 1 | 42.29 ± 3.83 | 20.90 ± 1.94 | 5.32 ± 0.17 |
| 2 | 223.53 ± 21.06 | 264.92 ± 6.12 | 348.40 ± 22.79 |
| 3 | 195.80 ± 12.40 | 552.34 ± 49.14 | 720.41 ± 19.76 |
| 4 | 179.43 ± 13.29 | 548.32 ± 28.06 | 876.23 ± 20.82 |
| 5 | 97.65 ± 5.14 | 539.38 ± 39.24 | 691.78 ± 28.48 |
| 6 | 34.76 ± 1.71 | 518.49 ± 33.54 | 636.73 ± 15.23 |
| 7 | 29.13 ± 1.82 | 441.13 ± 17.74 | 636.73 ± 51.80 |
| 8 | 28.73 ± 2.78 | 387.38 ± 28.10 | 574.54 ± 45.00 |
| 9 | 27.12 ± 1.21 | 260.60 ± 17.54 | 559.57 ± 31.80 |
| 10 | 20.60 ± 0.35 | 252.96 ± 19.36 | 461.82 ± 37.01 |

**Supplementary Table 2** Laccase activity from *Lenzites betulina* Han 851 grown on *Pinus tabuliformis*, *Firmiana platanifolia*, and a mixture by *Pinus tabuliformis* and *Firmiana platanifolia*.

| Day | Laccase of *Lenzites betulina* Han 851 (U/L) | | |
| --- | --- | --- | --- |
|  | *Pinus tabuliformis* | *Firmiana platanifolia* | A Mixture by *Pinus tabuliformis* and *Firmiana platanifolia* |
| 1 | 0.00 ± 0.00 | 2.01 ± 0.17 | 15.67 ± 0.52 |
| 2 | 0.00 ± 0.00 | 3.21 ± 0.17 | 59.17 ± 2.53 |
| 3 | 0.00 ± 0.00 | 46.92 ± 1.94 | 96.75 ± 1.38 |
| 4 | 8.54 ± 0.70 | 90.32 ± 5.19 | 136.23 ± 3.67 |
| 5 | 28.53 ± 2.45 | 158.23 ± 5.82 | 124.57 ± 3.47 |
| 6 | 36.57 ± 3.39 | 295.76 ± 12.71 | 113.62 ± 4.14 |
| 7 | 32.85 ± 1.09 | 309.72 ± 12.53 | 93.33 ± 2.34 |
| 8 | 31.85 ± 2.97 | 274.66 ± 8.72 | 86.90 ± 1.14 |
| 9 | 28.43 ± 0.97 | 230.16 ± 16.46 | 83.58 ± 0.76 |
| 10 | 22.91 ± 2.11 | 224.13 ± 19.37 | 66.91 ± 1.57 |

**Supplementary Table 3** Laccase activity from *Schizophyllum commune* Han 881 grown on *Pinus tabuliformis*, *Firmiana platanifolia*, and a mixture by *Pinus tabuliformis* and *Firmiana platanifolia*.

| Day | Laccase of *Schizophyllum commune* Han 881 (U/L) | | |
| --- | --- | --- | --- |
|  | *Pinus tabuliformis* | *Firmiana platanifolia* | A Mixture by *Pinus tabuliformis* and *Firmiana platanifolia* |
| 1 | 0.00 ± 0.00 | 0.00 ± 0.00 | 0.00 ± 0.00 |
| 2 | 0.00 ± 0.00 | 0.00 ± 0.00 | 0.00 ± 0.00 |
| 3 | 1.51 ± 0.00 | 0.90 ± 0.00 | 0.00 ± 0.00 |
| 4 | 0.00 ± 0.00 | 0.90 ± 0.00 | 0.00 ± 0.00 |
| 5 | 0.00 ± 0.00 | 1.21 ± 0.00 | 0.00 ± 0.00 |
| 6 | 0.00 ± 0.00 | 4.62 ± 0.35 | 0.00 ± 0.00 |
| 7 | 0.00 ± 0.00 | 5.22 ± 0.35 | 3.21 ± 0.17 |
| 8 | 0.00 ± 0.00 | 1.21 ± 0.00 | 3.32 ± 0.30 |
| 9 | 0.00 ± 0.00 | 0.30 ± 0.00 | 0.00 ± 0.00 |
| 10 | 0.00 ± 0.00 | 0.00 ± 0.00 | 0.00 ± 0.00 |

**Supplementary Table 4** Laccase activity from *Cerrena unicolor* Han 849 and *Schizophyllum commune* Han 881 grown on *Pinus tabuliformis*, *Firmiana platanifolia*, and a mixture by *Pinus tabuliformis* and *Firmiana platanifolia*.

| Day | Laccase of *Cerrena unicolor* Han 849 and *Schizophyllum commune* Han 881 (U/L) | | |
| --- | --- | --- | --- |
|  | *Pinus tabuliformis* | *Firmiana platanifolia* | A Mixture by *Pinus tabuliformis* and *Firmiana platanifolia* |
| 1 | 48.52 ± 3.78 | 206.95 ± 12.14 | 1.51 ± 0.00 |
| 2 | 58.17 ± 3.67 | 599.56 ± 12.45 | 58.87 ± 5.66 |
| 3 | 61.99 ± 3.02 | 622.36 ± 8.49 | 483.83 ± 5.69 |
| 4 | 118.24 ± 3.72 | 699.12 ± 0.76 | 491.66 ± 15.30 |
| 5 | 141.95 ± 5.82 | 778.78 ± 16.97 | 540.69 ± 19.37 |
| 6 | 202.73 ± 8.04 | 1373.12 ± 55.93 | 785.61 ± 37.51 |
| 7 | 219.41 ± 11.63 | 965.51 ± 11.29 | 490.26 ± 13.80 |
| 8 | 202.33 ± 15.56 | 887.88 ± 42.69 | 381.25 ± 12.53 |
| 9 | 182.94 ± 17.85 | 793.15 ± 12.66 | 366.49 ± 19.30 |
| 10 | 116.84 ± 1.25 | 556.66 ± 25.37 | 324.50 ± 9.69 |

**Supplementary Table 5** Laccase activity from *Lenzites betulina* Han 851 and *Schizophyllum commune* Han 881 grown on *Pinus tabuliformis*, *Firmiana platanifolia*, and a mixture by *Pinus tabuliformis* and *Firmiana platanifolia*.

| Day | Laccase of *Lenzites betulina* Han 851 and *Schizophyllum commune* Han 881 (U/L) | | |
| --- | --- | --- | --- |
|  | *Pinus tabuliformis* | *Firmiana platanifolia* | A Mixture by *Pinus tabuliformis* and *Firmiana platanifolia* |
| 1 | 14.47 ± 1.31 | 15.47 ± 0.97 | 82.38 ± 4.44 |
| 2 | 49.73 ± 2.58 | 156.92 ± 3.92 | 139.04 ± 2.42 |
| 3 | 52.14 ± 0.80 | 219.41 ± 4.25 | 150.59 ± 4.39 |
| 4 | 96.04 ± 3.93 | 289.03 ± 4.96 | 135.32 ± 12.16 |
| 5 | 44.20 ± 0.97 | 549.83 ± 12.42 | 131.61 ± 12.39 |
| 6 | 38.98 ± 0.46 | 350.81 ± 5.63 | 109.40 ± 9.08 |
| 7 | 38.08 ± 1.49 | 344.08 ± 10.58 | 183.34 ± 13.13 |
| 8 | 35.97 ± 1.91 | 340.16 ± 4.23 | 129.40 ± 2.82 |
| 9 | 37.77 ± 2.05 | 301.89 ± 4.22 | 121.06 ± 2.44 |
| 10 | 30.84 ± 0.46 | 283.50 ± 9.58 | 94.53 ± 2.44 |

**Supplementary Table 6** Laccase activity from *Cerrena unicolor* Han 849 and *Lenzites betulina* Han 851 grown on *Pinus tabuliformis*, *Firmiana platanifolia*, and a mixture by *Pinus tabuliformis* and *Firmiana platanifolia*.

| Day | Laccase of *Cerrena unicolor* Han 849 and *Lenzites betulina* Han 851 (U/L) | | |
| --- | --- | --- | --- |
|  | *Pinus tabuliformis* | *Firmiana platanifolia* | A Mixture by *Pinus tabuliformis* and *Firmiana platanifolia* |
| 1 | 24.01 ± 0.46 | 88.71 ± 6.19 | 0.00 ± 0.00 |
| 2 | 45.71 ± 2.63 | 574.24 ± 15.81 | 158.13 ± 14.46 |
| 3 | 51.64 ± 2.73 | 636.43 ± 21.83 | 210.57 ± 5.19 |
| 4 | 60.38 ± 2.93 | 691.08 ± 22.30 | 238.60 ± 3.31 |
| 5 | 44.00 ± 0.30 | 719.41 ± 30.77 | 316.76 ± 5.50 |
| 6 | 35.66 ± 1.25 | 766.02 ± 15.70 | 361.86 ± 16.35 |
| 7 | 33.86 ± 1.25 | 785.61 ± 69.52 | 387.18 ± 25.93 |
| 8 | 25.32 ± 2.09 | 839.51 ± 30.72 | 390.30 ± 12.89 |
| 9 | 23.01 ± 0.70 | 975.59 ± 47.66 | 349.21 ± 13.21 |
| 10 | 17.58 ± 0.35 | 1144.85 ± 34.97 | 328.31 ± 8.07 |

**Supplementary Table 7** Laccase activity from *Cerrena unicolor* Han 849, *Lenzites betulina* Han 851 and *Schizophyllum commune* Han 881 grown on *Pinus tabuliformis*, *Firmiana platanifolia*, and a mixture by *Pinus tabuliformis* and *Firmiana platanifolia*.

| Day | Laccase of *Cerrena unicolor* Han 849, *Lenzites betulina* Han 851 and *Schizophyllum commune* Han 881 (U/L) | | |
| --- | --- | --- | --- |
|  | *Pinus tabuliformis* | *Firmiana platanifolia* | A Mixture by *Pinus tabuliformis* and *Firmiana platanifolia* |
| 1 | 9.54 ± 0.46 | 9.34 ± 0.52 | 3.72 ± 0.35 |
| 2 | 44.71 ± 1.36 | 157.83 ± 4.11 | 70.42 ± 2.01 |
| 3 | 36.87 ± 1.42 | 276.17 ± 7.77 | 115.33 ± 4.35 |
| 4 | 32.35 ± 2.44 | 279.89 ± 9.51 | 143.66 ± 3.92 |
| 5 | 31.34 ± 1.38 | 305.51 ± 12.17 | 166.67 ± 5.52 |
| 6 | 31.14 ± 0.76 | 390.60 ± 2.76 | 274.46 ± 16.10 |
| 7 | 29.33 ± 1.14 | 521.00 ± 13.45 | 227.65 ± 4.91 |
| 8 | 28.23 ± 1.36 | 622.66 ± 6.02 | 201.43 ± 8.27 |
| 9 | 28.33 ± 0.80 | 664.66 ± 19.11 | 143.16 ± 6.23 |
| 10 | 26.82 ± 0.90 | 774.96 ± 13.79 | 60.38 ± 2.30 |
